# Prevalence of subclinical mastitis in Finnish dairy cows: changes during recent decades and impact of cow and herd factors

**DOI:** 10.1186/s13028-017-0288-x

**Published:** 2017-04-20

**Authors:** Heidi Hiitiö, Johanna Vakkamäki, Heli Simojoki, Tiina Autio, Jouni Junnila, Sinikka Pelkonen, Satu Pyörälä

**Affiliations:** 1 Department of Production Animal Medicine, Faculty of Veterinary Medicine, University of Helsinki, Paroninkuja 20, 04920 Saarentaus, Finland; 2 Veterinary Bacteriology Research Unit, Finnish Food Safety Authority Evira, Neulaniementie 4, 70210 Kuopio, Finland; 3 Oy 4Pharma Ltd., Mannerheimintie 44 A, 00260 Helsinki, Finland

**Keywords:** Prevalence, Bovine, Subclinical mastitis, SCC, Chronic subclinical mastitis

## Abstract

**Background:**

The dairy industry has undergone substantial structural changes as intensive farming has developed during recent decades. Mastitis continues to be the most common production disease of dairy cows. Nationwide surveys of mastitis prevalence are useful in monitoring udder health of dairy herds and to study the impact of structural changes on the dairy industry. This survey on bovine subclinical mastitis was the first based on cow composite milk somatic cell count (SCC) data from the Finnish national health monitoring and milk recording database. A cow with composite milk SCC ≥200,000 cells/ml in at least one of the four test milkings during the year was considered to have subclinical mastitis and a cow with composite milk SCC ≥200,000 cells/ml in three or in all four test milkings during the year to have chronic subclinical mastitis. The aim of the study was to determine the prevalence of subclinical mastitis and chronic subclinical mastitis in Finland in 1991, 2001 and 2010 and to investigate cow and herd factors associated with elevated SCC.

**Results:**

Prevalence of subclinical mastitis in Finland decreased over recent decades from 22.3% (1991) and 20.1% (2001) to 19.0% (2010). Prevalence of chronic subclinical mastitis was 20.4% in 1991, 15.5% in 2001 and 16.1% in 2010. The most significant cow and herd factors associated with subclinical mastitis or high milk SCC were increasing parity, Holstein breed, free-stalls with an automatic milking system and organic production. Milk SCC were highest from July to September. Main factors associated with chronic mastitis were increasing parity and Holstein breed.

**Conclusions:**

Prevalence of subclinical mastitis in Finland decreased over recent decades, the greatest change taking place during the first decade of the study. Prevalence of chronic subclinical mastitis significantly decreased from 1991. The most significant factors associated with both types of mastitis were increasing parity and Holstein breed, and for subclinical mastitis also free-stalls with automatic milking. National surveys on mastitis prevalence should be carried out at regular intervals to monitor udder health of dairy cows and to study the impact of the ongoing structural changes in the dairy industry to enable interventions related to udder health to be made when needed.

**Electronic supplementary material:**

The online version of this article (doi:10.1186/s13028-017-0288-x) contains supplementary material, which is available to authorized users.

## Background

The dairy industry has undergone structural changes during recent decades in many countries as the number of dairy herds has decreased but herd size substantially increased [1–3]. Simultaneously barn types and milking systems have changed, but nonetheless mastitis continues to be the most common and costly production disease of dairy cows [4]. In Finland, herd size and average milk yield have increased, while the number of dairy cows has declined [2]. The proportion of free-stalls has increased rapidly, especially those with automatic milking systems (AMS). The number of stalls with AMS (with one or more milking robots) increased from 0 in 1991 to near 600 in 2010 and continues to increase (currently about 950, personal communication, Esa Manninen, Valio Ltd., January 2016). Larger herds kept in free-stalls instead of tie-stalls, in addition to new milking technology, represent challenges for udder health management and may increase the risk for high milk somatic cell counts (SCC), as previously recognized [5, 6]. Up-to-date information on milk SCC and mastitis prevalence, as well as on factors affecting them, are useful to increase the efficiency of udder health management. Knowledge of prevalence trends provides feedback on control measures taken and improves guidance for future strategies.

The Finnish national health monitoring and milk recording system of dairy herds was initiated in the late 1970s and was fully constituted in 1982 [7]. Finland has a long history of nationally organized mastitis control programs, which have included regular surveys on mastitis prevalence [8–11]. Finnish prevalence studies published to date have been based on quarter milk samples and have focused on bacteriology, using quarter milk SCC ≥300,000 cells/ml of milk at least in one quarter of the cow to define mastitis. According to the studies, mastitis prevalence in Finland decreased from 47.8% (1988) to 37.8% (1995) [10]. In the most recent survey (2001), mastitis prevalence was 30.6% [11]. All previous surveys cited here use the general term mastitis, but based on the accepted definitions the focus has been on subclinical mastitis (SCM).

For herds contributing data to the Finnish national health monitoring and milk recording system, milk characteristics, including cow composite SCC for every lactating cow of each herd, have been recorded at least four times a year. Currently, the data cover approximately 81% of Finnish dairy cows. To date, the Finnish national health monitoring and milk recording database has been exploited only for udder health management on farms, but not comprehensively at the national level. The aim of this study was to determine the prevalence of SCM and chronic subclinical mastitis (CSCM) in Finland in 1991, 2001 and 2010 by analyzing national cow composite SCC data and using a threshold of ≥200,000 cells/ml. Cow and herd factors affecting SCC and their associations with SCM and CSCM were studied.

## Methods

### Data collection

Somatic cell counts data were analyzed from the Finnish national health monitoring and milk recording database from 1991 (cows, n = 122,403, herds, n = 20,346), 2001 (cows, n = 337,335, herds, n = 13,749) and 2010 (cows, n = 273,012, herds, n = 7640). The proportion of herds in the system increased from 62% in 1991 to 81% 2010 (ProAgria Agricultural processing center). Sampling herds associated with the Finnish national health monitoring and milk recording database is carried out at least five times a year per herd and includes all lactating cows at least 2 days in milk (DIM). All samples were cow composite samples. In the study, the first sample result for each cow from each quartile (January–March, April–June, July–September, October–December) was selected (maximum of four results per cow) to maximize the number of cows, including from 1991, when sampling was not as frequent as now. If the cow was culled or otherwise removed from the herd or dried-off, those results available for the year were included.

Samples were collected in 30 ml plastic tubes with preservative (bronopol), using specific sampling devices during milking, or by automatic sampling devices on milking robots. The samples were sent to pre-assigned regional laboratories and SCC was determined with a fluoro-optical method using Fossomatic™ FC (FOSS Ltd., Hillerød, Denmark). Guidelines for sampling were similar over the years, but sampling has become automated in free-stalls with AMS. The gathered data included information for individual cows—breed, age, parity, cow composite SCC and total quantity of milk produced during the first 305 days lactation period for each cow. Herd level information was provided for each cow: production type, type of stall, herd size, milking system, and average annual milk SCC and milk yield of the herd (Table 1).

**Table 1:** Description of the variables used in the study

Analysis	Variable	Description of the variable
Prevalence of SCM (subclinical mastitis) and CSCM (chronic subclinical mastitis)		
	Breed	Holstein, Ayrshire, Other. Group “other breeds” includes Finncattle, Jerseys and their crossbreds and single cows of some other breeds
	Herd size	Average herd size *(continuous variable)*. The average herd size is calculated as follows: (Days in feeding_cow1_^a^ + Days in feeding _cow2_….) divided by 365 days (or 366 days if leap year) i.e. the total amount of days within the year of recording
	Milk yield	305 days milk yield from each cow primiparous period *(continuous variable)*
	Parity	Parity 1 to ≥4 *(categorical variable)*
	Production type	Organic, conventional
	Quartile^b^	Quartile of the year—Jan–Mar, Apr–Jun, Jul–Sept, Oct–Dec
	Region	Geographical region of Finland—South, West, North, East. Every herd belonged to one of the 22 regional ProAgria Agricultural units in study years of 1991, 2001 and 2010. These units were divided according to province borders (in 2012) into four geographic regions
	Type of the stall^c^	Tie-stall, free-stall, free-stall with AMS (automatic milking system)
	Year	Year of the data recording—1991, 2001, 2010
SCC		
*Cow level*	Breed	Holstein, Ayrshire, Other. Group “other breeds” includes Finncattle, Jerseys and their crossbreds and single cows of some other breeds
	Herd size	Average herd size *(continuous variable)*. The average herd size was calculated as follows: (Days in feeding_cow1_^a^ + Days in feeding_cow2_….) divided by 365 days (or 366 days if leap year) i.e. the total amount of days within the year of recording
	Milk yield	305 days milk yield from the first lactation period *(continuous variable)*
	Parity	Parity 1 to ≥4 *(continuous variable)*
	Production type	Organic, conventional
	Quartile	Quartile of the year—Jan–Mar, Apr–Jun, Jul–Sept, Oct–Dec
	Region	Geographical region of Finland—South, West, North, East. Every herd belonged to one of the 22 regional ProAgria Agricultural units in study years of 1991, 2001 and 2010. These units were divided according to province borders (in 2012) into four geographic regions
	Type of stall^b^	Tie-stall, free-stall, free-stall with AMS (automatic milking system)
	Year	Year of the data recording—1991, 2001, 2010
*Herd level*	Herd size	Average herd size *(continuous variable)*. The average herd size was calculated as follows: (Days in feeding_cow1_^a^ + Days in feeding_cow2_….) divided by 365 days (or 366 days if leap year) i.e. the total amount of days within the year of recording
	Herd milk yield	Average annual milk yield of the herd *(continuous variable)*. Milk yield of the herd was calculated as follows: (Milk kg of the year_cow 1_ + Milk kg of the year_cow 2_ + Milk kg of the year_cow 3_ + Milk kg of the year cow 4..)/average herd size
	Average parity, herd	Average parity of the cows in the herd *(continuous)*
	Region	Geographical region of Finland—South, West, North, East. Every herd belonged to one of the 22 regional ProAgria Agricultural units in study years of 1991, 2001 and 2010. These units were divided according province borders (in 2012) into four geographic regions
	Type of the stall^b^	Tie-stall, free-stall, free-stall with AMS (automatic milking system)
	Year	Year of the data recording—1991, 2001 and 2010

^a^ Recording of feeding days starts from the first calving or the date that the cow enters the herd and ends when it is culled or otherwise removed from the herd. (Personal communication, specialist Juho Kyntäjä, ProAgria Agricultural processing center, August 2015)

^b^ Not included in CSCM model

^c^ Data available only for years 2001 and 2010

Cows with a cow composite milk SCC of ≥200,000 cells/ml in at least one of the four test milkings for the year were defined as having subclinical mastitis [13]. SCM refers to udder inflammation (increased milk SCC) that continues for some period of time but ceases by the next sampling. Cows with a cow composite milk SCC ≥200,000 cells/ml in three or all four test milkings during the year were recognized as having chronic subclinical mastitis. CSCM refers to udder inflammation (increased milk SCC) that continues for a long period of time. Only cows with test results from every quartile of the year were included in the analyses of CSCM (n = 100,261 cows in 1991, 220,354 in 2001 and 180,557 in 2010).

### Descriptive analysis

First, the data were checked and evaluated for outliers and missing values (Microsoft Office, Excel 2010). The most frequent error was a letter or null instead of a consistent value. For some proportion of cows all information was not available, and the number of these cows for each variable is shown in the descriptive data (Tables 2, 3). The number and proportion of the cows with SCM were calculated for the year, annual quartile and for the following subgroups: parity (1 to ≥4), breed (Ayrshire also known as Nordic Red, Holstein and others), type of the stall (tie-stall, free-stall or free-stall with AMS), herd size (<20, 20–60, > 60 cows), average annual milk yield of the herd (<7500, 7500–9500 and > 9500 kg), geographical region of Finland (South, West, North, East) and production type (organic or conventional). The numbers of Finncattle, Jerseys and other breeds were so low that they were grouped together as ‘other breeds`. The cows with CSCM were assessed accordingly, except for annual quartile.

**Table 2:** Descriptive data of cows with subclinical mastitis in Finland in 1991, 2001 and 2010

	Year					
	1991		2001		2010	
	n, cows^a^	%, cows with SCM^b^	n, cows^a^	%, cows with SCM^b^	n, cows^a^	%, cows with SCM^b^
Parity						
1	24,791	12.2	104,995	16.2	85,532	14.3
2	25,896	14.5	89,197	17.1	75,156	15.4
3	25,737	23.8	61,749	22.2	52,769	21.8
4	19,642	28.6	37,919	25.4	31,082	25.9
5	11,539	31.9	20,405	27.4	15,833	28.2
6	6235	35.0	10,239	27.6	7110	29.3
7	3150	36.4	4669	30.6	3005	31.9
8	1544	37.7	2034	29.8	1329	31.2
≥9	1224	40.0	1495	31.7	1196	35.5
n/a^d^	2645		4633		0	
Breed						
Ayrshire	99,070	21.4	247,736	19.3	175,583	18.0
Holstein	21,776	26.3	85,704	22.3	93,644	20.8
Others^c^	1557	26.9	3872	21.7	3522	18.9
n/a^d^	0		23		263	
Type of the stall						
Tie-stall	0	0	82,688	20.5	108,824	18.7
Free-stall	0	0	9322	21.1	49,837	20.9
Free-stall (AMS)	0	0	0^e^	0^e^	21,712	22.6
N/a^d^	122,403		245,325		92,639	
Herd size, number of cows						
<20	99,832	22.0	150,611	19.8	49,446	17.5
20–60	21,504	23.6	174,433	20.1	158,661	18.6
>60	584	27.4	7532	23.5	59,430	21.4
n/a^d^	483		4759		5475	
Average milk yield (kg/year/herd)						
<7500	109,083	22.8	116,053	22.8	26,109	23.8
7500–9500	13,048	18.7	195,811	19.0	161,262	19.8
>9500	243	15.2	25,361	16.6	85,618	16.5
n/a^d^	29		110		23	
Production type						
Organic	15	13.3	6300	22.7	4300	22.2
Conventional	122,386	22.3	331,035	20.1	268,450	18.9
n/a^d^	2		0		262	
Total	122,403	22.3	337,335	20.1	273,012	19.0

^a^ Total number of the cows of the study year

^b^ Cows with a composite milk SCC ≥200,000 cells/ml in at least one of the four test milkings of the year

^c^ Including Finncattle, Jerseys and other breeds

^d^ Information not available

^e^ Three AMS barns in 2001 were excluded from statistical calculations

**Table 3:** Descriptive data of cows with chronic subclinical mastitis in Finland in 1991, 2001 and 2010

	Year					
	1991		2001		2010	
	n, cows^a^	%, cows with CSCM^b^	n, cows^a^	%, cows with CSCM^b^	n, cows^a^	%, cows with CSCM^b^
Parity						
1	9676	7.8	41,444	11.3	39,148	10.5
2	24,582	12.2	73,130	13.3	59,752	13.0
3	24,041	20.3	47,476	16.7	39,401	18.2
4	18,206	25.4	27,959	19.3	22,286	21.9
5	10,538	29.5	14,563	20.9	11,136	24.2
6	5699	31.1	7190	21.6	4934	26.0
7	2857	32.6	3233	22.8	2112	27.9
8	1387	35.8	1421	22.4	886	26.1
≥9	1101	35.9	1063	25.3	843	33.9
N/a^d^	2174		2875		59	
Breed						
Ayrshire	81,185	19.1	162,484	14.5	116,443	14.6
Holstein	17,796	25.9	55,415	18.6	61,879	18.8
Others^c^	1280	24.4	2455	15.8	2235	15.3
N/a^d^	0		0		0	
Type of the stall						
Tie-stall	0	0	74,974	15.0	99,417	14.8
Free-stall	0	0	8444	16.1	45,098	16.6
Free-stall (AMS)	0	0	0^e^	0^e^	19,595	21.3
N/a^d^	100,261		136,936		16,447	
Herd size, cows						
<20	81,533	19.8	98,895	15.1	33,118	14.0
20–60	17,838	22.4	113,134	15.6	105,203	15.4
>60	508	32.1	4847	19.7	38,738	19.8
N/a^d^	382		3478		3498	
Average milk yield (kg/year/herd)						
<7500	89,100	20.9	73,763	18.0	14,635	19.5
7500–9500	10,954	16.5	129,343	14.4	105,937	16.4
>9500	207	15.5	17,248	13.1	59,985	14.6
N/a^d^	0		0		0	
Production type						
Organic	14	14.3	4028	18.8	2933	18.4
Conventional	100,247	20.4	216,326	15.5	177,624	16.0
N/a^d^	0		0		0	
Total	100,261	10.4	220,354	15.5	180,557	16.1

^a^ Total number of the cows of the study year

^b^ Cows with a composite milk SCC ≥200,000 cells/ml in three or all four of the test milkings of the year

^c^ Including Finncattle, Jerseys and other breeds

^d^ Information was not included to the original recording (missing value)

^e^ Three AMS barns in 2001 were excluded from statistical calculations

### Statistical methods

The associations of the SCC of the cows and explanatory factors were analyzed with linear mixed models. Logarithmic transformation was used to normalize the SCC-distribution, thus LnSCC was used as the response variable in the model. The factors (Table 1) were first modeled separately, so that only the year, quartile, the defined factor and the interaction between the factor and year were included in the model as fixed factors, herd as a random effect and cow as a subject effect. Secondly, a multivariable mixed effects linear mixed model was fitted, where all statistically significant explanatory factors and their significant interactions with year were included in the same model. The statistical significance was determined based on Type III tests for fixed effects. This was done to assess all the factors simultaneously and to exclude possible confounding effects. The effects of the explanatory variables were quantified with least square means and 99.9% confidence limits (CL) (within and between group), calculated from the final multivariable model.

The effects of the variables presented in Table 1 on LnSCC of the herd were analyzed using ANOVA models. The fitted univariable models included the year, the defined factor and the interaction term of the factor and year as fixed factors. In addition, all significant explanatory factors were included in a multivariable ANOVA. The effects of the explanatory variables were quantified with least square means and 99% CL, calculated from the final multivariable model.

Effects of the same explanatory factors (Table 1) on the proportion of cows with SCC ≥200,000 cells/ml were studied with mixed effects logistic regression models, using data from all four quartiles. A similar analysis strategy (univariable and multivariable models), and the same fixed and random effects, were included as described for the mixed effects linear regression models. The statistical significance was determined based on Type III tests for fixed effects.

Similar mixed effects logistic regression models were constructed for the proportion of cows with CSCM. Explanatory factors (Table 1) were included in the model based on data from the first quartile. The definition of CSCM prohibits the possibility of studying effects of season, thus no seasonal effects were included in the models. Differences among the groups were quantified with odds ratios (OR) and their 99.9% CL.

Two different definitions of statistical significance were used. For herd level analyses, a probability level of <0.01 was considered statistically significant. In analyses based on individual cows, p < 0.001 was considered statistically significant. The significance limits were kept low because the datasets were large (they included data for most dairy cows in Finland). This led to very precise estimations of the effects and therefore the usual limits (e.g. p < 0.05) were not suitable for applying to the results. All p-values were 2-sided and not adjusted for multiple testing. Some of the investigated factors were only measured from the 2001 and 2010 data (see Table 1). Thus, the final statistical models were constructed both for the full data and for a subset of the data including only those for 2001 and 2010. The results of the models including years 2001 and 2010 are provided in the supplementary data.

All statistical analyses were done using SAS System for Windows, version 9.3 (SAS Institute Inc., Cary, NC, USA).

## Results

The total number of cows included in the study and numbers in different subgroups for each study year, in addition to the proportion of cows with SCM, are presented in Table 2. The number of cows included in the CSCM investigation and the proportion (%) of cows with CSCM are presented in Table 3. The figures are given in total and in subgroups (Table 3).

### Prevalence of SCM and associated factors

**Fig. 1:**
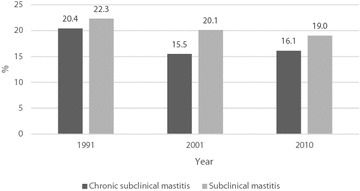
Prevalence of subclinical mastitis (SCM) and chronic subclinical mastitis (CSCM) in years 1991, 2001 and 2010. Prevalence of subclinical mastitis (SCM) and chronic subclinical mastitis (CSCM) of dairy cows in the Finnish national health monitoring and milk recording system in 1991 (SCM n = 27,296 and CSCM n = 10,427), 2001 (SCM n = 67,804 and CSCM n = 34,155) and 2010 (SCM n = 51,872 and CSCM n = 29,070). A cow was assumed to have SCM in the study if it had a composite milk SCC ≥200,000 cells/ml in at least one of the four test milkings for each study year. A cow was assumed to have CSCM if it had a composite milk SCC ≥200,000 cells/ml in three or all four test milkings during the study year

**Fig. 2:**
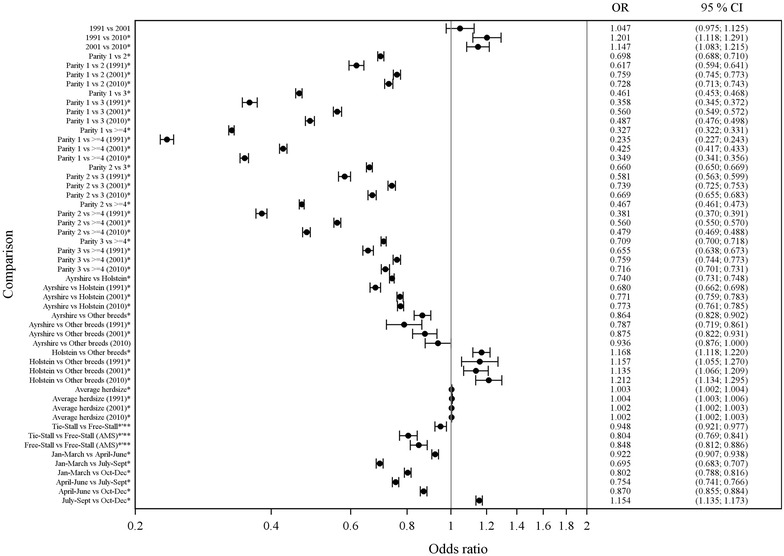
Comparison of the impact of different factors on the prevalence of subclinical mastitis (SCM). Mixed effects logistic regression model of SCM and related factors. SCM was defined as cow composite milk SCC ≥200,000 cells/ml in one test milking of the year (observations used 2,050,362). Interactions between years and different variables are also presented. Results of the effect of different regions of Finland are described in the text. *OR* odds ratio, *95% CL* confidence limit. *Statistically significant. **Type of stall estimations only from years 2001 and 2010

In 1991, the prevalence SCM (22.3%) was higher than in 2001 (20.1%) and in 2010 (19.0%, Fig. 1). The risk for SCM increased with increasing parity of the cow (Fig. 2), but in every parity group odds ratio (OR) for SCM was lower in 2010 than in 2001. Ayrshire cows, among other breeds, had a lower OR for SCM than Holsteins (Fig. 2). The milk yield from the first 305 days lactation for the primiparous cow did not affect the risk for SCM and was excluded from the final model.

According to the final model of logistic regression analyses, herd size had only a minimal increasing effect on SCM (Fig. 2). In 2001 and 2010 data, tie-stalls and free-stalls had lower OR for SCM than free-stalls with AMS (Additional file 1).

The risk for SCM was lower in East Finland compared with West (OR 0.863, CL 0.851–0.875) and South (OR 0.903, CL 0.887–0.918) and in North Finland compared with West (OR 0.860, CL 0.849–0.877) and South (OR 0.900, CL 0.886–0.913). The risk for SCM was lower during the first quartile, from January to March than during the other quartiles (Fig. 2). The highest risk for SCM was during July–September compared with the three other quartiles (Fig. 2).

### Prevalence of CSCM and associated factors

**Fig. 3:**
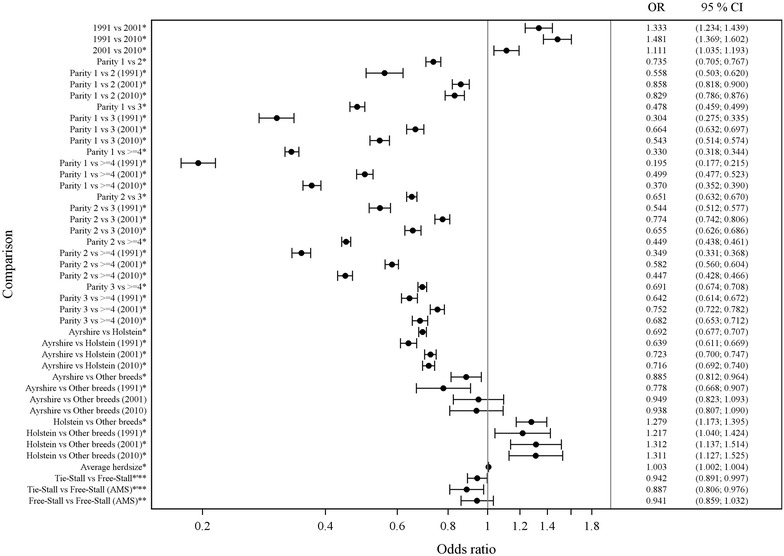
Comparison of the impact of different factors on the prevalence of chronic subclinical mastitis (CSCM). Mixed effects logistic regression model of CSCM and related factors. CSCM was defined as cow composite milk SCC ≥200,000 cells/ml in three or four test milkings of the year (observations used 499,376). Interactions between years and different variables are also presented. Results of the effect of different regions of Finland are described in the text. *OR* odds ratio, *95% CL* confidence limit. *Statistically significant. **Type of stall estimations only from years 2001 and 2010

The prevalence of CSCM was higher in 1991 (20.4%) than in 2001 (15.5%) and in 2010 (16.1%, Figs. 1, 3). The risk for CSCM in 2001 was higher than in 2010 (Fig. 3), despite that the proportion of cows with CSCM was slightly lower in 2001 compared with 2010. The OR for CSCM increased with increasing parity (Fig. 3). The effect of breed on CSCM was similar as on SCM: Ayrshire cows had lower OR for CSCM than Holstein cows (Fig. 3). Differences in ORs between these two breeds decreased over the two decades.

Herd size had only a minor increasing effect on CSCM (Fig. 3). The effect of different stall-types on the risk of having CSCM was similar than with SCM (Fig. 3). The regional associations with CSCM were included in the same mixed effects logistic regression model (Fig. 3). The OR for CSCM was lower in West compared with East (OR 0.872, CL 0.828–0.917) and North (OR 0.815, CL 0.783–0.847) of Finland.

### SCC results at cow level

Average cow composite milk SCC values with standard deviations are presented in Table 4, grouped by breed, stall-type, production type and region. Average arithmetic milk SCC decreased from 209,200 cells/ml in 1991 to 192,500 cells/ml in 2001 and 192,000 cells/ml in 2010 (Table 4).

**Table 4:** Cow composite milk somatic cell count (SCC) (mean, SD) and related factors in Finland in 1991, 2001 and 2010

	1991		2001		2010	
	SCC × 10^3^	SD	SCC × 10^3^	SD	SCC × 10^3^	SD
Ayrshire	201.8	425.3	186.6	467.8	182.0	472.8
Holstein	240.5	452.5	209.3	483.4	210.7	506.7
Others^a^	245.8	488.8	208.2	521.8	199.8	522.9
Tie-stall	n/a^b^	n/a^b^	186.5	457.3	179.1	450.9
Free-stall	n/a^b^	n/a^2^	197.8	488.1	200.2	499.2
Free-stall (AMS)	n/a^b^	n/a^b^	n/a^b^	n/a^b^	238.8	599.4
Organic production	n/a^b^	n/a^b^	n/a^b^	n/a^b^	213.9	539.9
Conventional production	209.2	431.4	192.0	471.0	191.7	484.5
South	217.9	460.6	206.6	510.9	204.5	526.4
West	219.8	456.0	206.2	508.4	200.2	499.8
East	194.9	393.5	177.7	425.1	180.4	455.2
North	202.6	411.7	178.0	432.2	182.9	463.4
Total	209.2	431.4	192.5	472.5	192.0	485.5

1991 n = 122,403, 2001 n = 337,335 and 2010 n = 273, 012

^a^ Including Finncattle, Jerseys and other breeds

^b^ Information not available

Average cow SCC values were lowest in primipara and increased with increasing parity (Table 5). Milk SCC of cows in each parity group increased during the study period (Table 5). Ayrshire cows had the lowest SCC and Holsteins the highest (Table 5), but SCC of all breed groups decreased from 2001 (Table 5).

**Table 5:** Estimated effects of different factors on cow composite milk SCC (somatic cell count) in Finland

Factor				1991			2001			2010		
	99.9% CI			99.9% CI			99.9% CI			99.9% CI		
	Estimate	Lower	Upper	Estimate	Lower	Upper	Estimate	Lower	Upper	Estimate	Lower	Upper
Year				72.4	61.1	85.8	82.1	81.1	83.1	74.7	73.7	75.8
Number of parity												
1	53.1	50.1	56.2	46.1	38.9	54.7	53.3	52.5	54.1	73.8	72.9	74.7
2	66.4	62.7	70.3	61.2	51.7	72.6	64.8	63.9	65.7	93.0	91.8	94.2
3	88.3	83.4	93.5	86.4	72.8	102.4	85.6	84.3	86.9	112.6	95.0	133.6
≥4	108.9	102.9	115.3	60.9	60.1	61.7	108.9	107.5	110.4	105.3	103.8	106.9
Breed												
Ayrshire	69.3	65.4	73.3	63.8	53.8	75.6	76.1	75.3	76.8	68.5	67.7	69.3
Holstein	86.0	81.2	91.0	82.5	69.6	97.8	91.8	90.9	92.8	83.9	82.8	84.9
Other breeds	74.6	70.3	79.2	72.2	60.7	85.8	79.3	77.2	81.4	72.6	70.6	74.6
Type of production												
Organic production	74.2	66.2	83.1	61.3	43.6	86.0	87.1	85.3	88.8	76.4	74.7	78.3
Conventional production	78.5	78.0	79.0	85.6	84.3	86.8	77.5	76.8	78.1	73.0	72.4	73.7
Region												
East	72.6	68.5	76.8	67.0	56.5	79.4	79.7	78.7	80.8	71.6	70.5	72.6
North	72.3	68.3	76.5	72.2	60.9	85.6	75.0	74.0	76.0	69.8	68.8	70.8
South	78.9	74.5	83.6	73.9	62.4	87.7	85.8	84.6	87.0	77.4	76.2	78.6
West	81.9	77.3	86.7	76.9	64.8	91.1	88.6	87.5	89.8	80.5	79.4	81.7

The estimated effects (multivariate ANOVA model) of the different pre-determined factors on composite milk SCC of cows included in the Finnish National health monitoring and milk recording system in 1991, 2001 and 2010 (observations used 2,050,362). Interactions between years and variables are presented separately in the columns. All the included factors were tested statistically significant with Type III tests for fixed effects

Effect of the stall type was studied only in 2001 and 2010 because the number of free-stalls was marginal in 1991 (Additional file 2). Cows in tie stalls had the lowest SCC estimates (77.2; CI 76.2–78.1) and cows in free stalls with AMS the highest (94.5; CI 93.0–95.9); estimates for cows in free stalls with milking parlors were between the two other stall types (80.3; CI 79.3–81.4; Additional file 2).

Cows in organic herds had higher SCC compared with cows in conventional production in years 2001 and 2010 (Additional file 2).

Milk yield of the first 305 days lactation period of the cow did not affect milk SCC and was excluded from the final model. The overall improvement of the cow composite milk SCC during the studied years can be calculated by summarizing the estimates. For example, a primiparous cow of Ayrshire breed from an organic herd in the West of Finland would have 26,400 cells/ml lower milk SCC in 2010 than in 1991. Similarly, a Holstein cow in her 4th lactation from a conventional herd in the West of Finland would have 14,900 cells/ml lower milk SCC in 2010 than in 1991.

### SCC results on herd level

Average cow composite milk SCC of dairy herds in Finland was higher in 1991 than in 2001 and 2010 (Table 6). For every 100 kg of milk produced in the herd, a slight increase in the average milk SCC was noted and this increase was highest in 2010 (Table 6).

**Table 6:** Estimated effects of different factors on average herd milk SCC (somatic cell count) in Finland

Comparison	99.9% CI			p value
	Estimate	Lower	Upper	
1991 vs. 2001	1.246	1.121	1.385	<0.001
1991 vs. 2010	1.630	1.437	1.849	<0.001
2001 vs. 2010	1.308	1.147	1.492	<0.001
Herd milk yield (100 kg)	0.990	0.989	0.990	<0.001
Herd milk yield (100 kg, 1991)	0.987	0.986	0.988	<0.001
Herd milk yield (100 kg, 2001)	0.989	0.988	0.990	<0.001
Herd milk yield (100 kg, 2010)	0.993	0.992	0.994	<0.001
Average herd size	1.007	1.006	1.008	<0.001
Average herd size (1991)	1.010	1.008	1.012	<0.001
Average herd size (2001)	1.007	1.006	1.009	<0.001
Average herd size (2010)	1.004	1.003	1.005	<0.001
South vs. West	1.011	0.987	1.035	0.236
South vs. North	1.118	1.089	1.148	<0.001
South vs. East	1.100	1.074	1.127	<0.001
West vs. North	1.106	1.083	1.129	<0.001
West vs. East	1.088	1.069	1.108	<0.001
North vs. East	0.984	0.964	1.005	0.051

The estimated effects (multivariate ANO VA model) of the dif ferent pre-determined factors on the average milk SCC of herds included in the Finnish national health monitoring and milk recording system in 1991, 2001 and 2010 (observations used 41,503). Interactions between years and variables are presented on separate lines. All the included factors were tested statistically significant with Type III tests for fixed effects

Average cow composite milk SCC for the herd increased with increasing herd size. According to our estimates, every cow added to the herd would increase milk SCC of the herd by approximately 1000 cells/ml. The effect of the increasing number of cows in the herd was smaller in 2010 than in 1991 or 2001 (Table 6).

Free-stall herds with or without AMS were associated with a higher average milk SCC than tie-stall herds (Additional file 3). Location of the herd in the South or West of Finland was also associated with a higher average milk SCC of the herd. Average parity of the herd did not affect milk SCC of the herd in the aggregated data, but average parity data only from 2001 and 2010 had a small effect (Additional file 3).

## Discussion

### Prevalence of SCM

The prevalence of SCM based on cow composite milk SCC has decreased in Finland during the past two decades, in particular during the first decade from 1991 to 2001. This may be due to a long-term national strategy to manage mastitis. Indexes related to udder health were introduced in the breeding programs of dairy cows. A working group of the Finnish Ministry of Agriculture and Forestry established during the late 1980s initiated this activity. Advisory services and training were provided to dairy farmers, but probably the most effective measure was implementing quality-based milk pricing. Consequently, prevalence of mastitis decreased during the next 20 years as seen in the successive national mastitis surveys [8–11]. At the same time, consumption of antibiotic intramammary products has decreased, which indicates that combatting SCM has not relied on antibiotic treatments [12].

Surveys are not directly comparable because methods and definitions differ, as do sampling schemes, which here comprised four samples during 1 year while the previous studies were based on a single sampling at one time point. However, based on the present study, though effects established were rather small, the same downward trend in the prevalence of SCM previously noted in Finland seems to continue.

Another factor which has had an impact on the udder health of Finnish dairy cows is their decreasing parity. In 1990, the average age of a dairy cow removed from the herd was 5.3 years, but in 2010 it was only 5.1 years [13]. Average parity of the herd decreases because of culling of the older cows and increasing the number of heifers after enlargement of the herd. The most common reasons for culling dairy cows in Finland are mastitis and fertility problems [13]. Premature culling of cows causes economic losses and addressing this problem is one of the biggest challenges for Finnish dairy farms [14].

During the twenty first century, general management of dairy herds as well as barn design and milking technology, improved in Finland, which also has contributed to the improved udder health reported in this study. The positive development of udder health of dairy cows contributes to the low SCC of the bulk milk in Finland. The geometric mean of bulk milk SCC in Finland has been under 150,000 cells/ml for 20 years, and it is the lowest in the European Union. However, although bulk milk SCC is related to udder health of the herd, it is not an accurate indicator of mastitis prevalence [15]. Milk from cows with high SCC is often separated from bulk milk, to maintain milk SCC in the premium class.

Prevalence and incidence of clinical and subclinical mastitis have been studied widely, but most published surveys have been based on random or ‘convenience’ samples [16–20]. Definitions used for mastitis also greatly differ among studies. To our knowledge, national dairy cow databases, covering the majority of dairy cows in a country, are available in Norway, Sweden and Denmark [7]. Similar national registers and the same definition of mastitis have been agreed on by the Committee for Milk Quality of the Nordic Dairy Organizations (NMSM) (personal communication, Laura Kulkas, Valio Ltd., December 2015). Because of the rather similar structure of dairy industry and the common definitions for mastitis, the present results could be applicable and used as a reference at least in the Nordic countries. In Sweden, the estimated SCM prevalence in 2012 using the SCC threshold of ≥200,000 cells/ml was 25.7% (personal communication, Håkan Landin, Växa Sverige, 2013), in Norway approximately 21.0% (personal communication, Olav Østerås, TINE advisory service, Norway, 2013), and in Denmark 26.0% [21]. Prevalence of mastitis in large Estonian herds, using a similar definition, was reported to be as high as 52.7% [22]. In a study comprising a random sample of large herds represented equally from all regions of the Netherlands, mean herd prevalence of SCM using the same SCC threshold as here was 12.8% in primipara and 27.1% in multipara [16]. Compared with figures presented in some other studies, the Finnish SCM prevalence of 19.0% in 2010 seems relatively low. Comparing mastitis prevalence studies from different countries or regions is however difficult due to different data collection protocols and study design. In addition, for instance, parity distribution of the cows included in the study should be considered.

### Prevalence of CSCM

This was the first Finnish study reporting the prevalence of CSCM, i.e. proportion of cows with milk SCC chronically ≥200,000 cells/ml. The prevalence of CSCM decreased over 20 years from 20.4% (1991) to 16.1% (2010). It should be kept in mind that only cows that had all four sample results available for the year were included. It is possible that a considerable proportion of cows with CSCM were lost due to culling during the study year, i.e. the prevalence reported here may be underestimated. The prevalences of CSCM were not very much lower than those of SCM, indicating that many cows with CSCM actually have long-term udder health problems.

No published data are available on the proportion of cows with CSCM from countries other than Finland. In Sweden in 2012 the estimated figure was comparable with ours, 16.0% (personal communication, Håkan Landin, Växa Sverige, 2013). In our study, the factors associated with CSCM were Holstein breed, increasing parity and free stalls. In large herds producing high daily milk volumes, usually in free stalls, bulk milk SCC may be maintained more easily in the premium class (in Finland bulk milk SCC <250,000 cells/ml) without treatment or discarding milk from cows with high SCC due to SCM. The slight increase in the proportion of cows with CSCM during the past 10 years may be related to the extensive enlargement of the herds with cows of unknown udder health status. The lower odds for CSCM in 2010, despite the higher prevalence, could be due to the better management and environment of the cows in the new free stalls.

Our definition of CSCM was novel and based on the results of four samples during each study year that were available for the study. The real udder health status of the CSCM cows remains unknown as cows can have truly persistent (chronic) infections or be re-infected. After all, the only reliable method for defining an intramammary infection as chronic would be repeated sampling of the mammary quarter over a long period and strain-typing of the isolated organisms to confirm the presence of the same infecting agent in the quarter. Here, the time between sampling of the cow was considered long enough for a possible cure from mastitis after the previous sampling, which would indicate SCM in this study. More than four samples during the study year would have provided more precise information on the udder health of the cows.

### Cow related factors associated with SCC, SCM and CSCM

Parity increased milk SCC and was associated with a higher OR for both SCM and CSCM. Our result supports the results from many previous studies [23, 24]. Mechanisms underlying this phenomenon are not fully understood, but could be related to the impairment of leukocyte functions with increasing age of the cow [25]. In principle, no other factor has been defined as an explicit cause of elevated milk SCC than intramammary infection [15]. The stress of subsequent lactation periods on the udder tissue and changes in udder conformation and depth increase exposure to intramammary infections [26]. Callosity of the teat end has also been recognized as a risk factor, and tends to increase with increasing parity [27].

Breed was another major factor related to SCC: Holsteins had significantly higher average SCC and higher OR for SCM and CSCM than Ayrshires. This result agrees with previous studies in which the Holstein breed was shown to be more susceptible to mastitis [19, 28–30]. The proportion of Holstein cows has continuously increased in Finland: in 1991 Holsteins represented 17.8% and Ayrshires 80.9% of dairy cows, but in 2010 the figures were 34.3 and 64.4%. Udder health improved among both breeds during the study period: the prevalence of SCM decreased in Holsteins from 26.3% (1991) to 20.8% (2010) and in Ayrshires from 21.4 to 18.0%, respectively. However, milk SCC increased in every parity group from 1991 to 2010, which is a reason for some concern. Udder health has been included as one of the most important factors for dairy cow breeding in Finland. Indexes like SCC, milking speed, leakage and the structure of the udder in daughter evaluation of the AI bulls, have been used in Finland for several decades. Cases of veterinary treated mastitis are also recorded and included in the breeding indexes. A possible threat in the future may be represented by increased use of genetic material from global breeding companies, which have no similar health data available. We assumed that the increase in SCC could be related to the increased milk production, but at least the milk yield for first 305 days lactation of the cow did not affect the SCC of the same cow.

The positive association between mastitis and milk yield is well established [31, 32]. Our results agree with earlier studies, but the effect we established was just moderate, yet statistically significant. Every 100 kg increase in the annual milk yield of a herd increased the average SCC of the herd by approximately 1000 cells/ml. This indicates that if the annual milk yield of a herd would increase from 10,000 to 12,000 kg, milk SCC of the herd would increase by 20,000 cells/ml, despite the year. In our study, high milk yield for the first 305 days had no impact on the milk SCC of the cow. Association of the milk yield and prevalence of SCM and CSCM was also very low (Tables 5, 6). High production herds with are usually managed expertly and breeding of the animals is systematic, which should improve udder health. In well-managed herds also treatment of mastitis is diagnosis-based and more efficient, which supports control of mastitis [33]. The dilution effect of the high milk yield on milk SCC may also play a role [31]. The only moderate increase of milk SCC seen here could be partly due to that phenomenon.

Cow composite milk SCC was higher for organic herds than conventional herds. A cow in an organic herd had in average 4300 cells/ml higher milk SCC than a cow in a conventional herd. This difference decreased during the study period, and in 2010 it was 3400 cells/ml. The proportion of cows with SCM was higher in organic herds (22.3%) compared with conventional herds (18.9%), which is consistent with the results on cow composite milk SCC. Results from previous studies comparing organic vs. conventional herds have been controversial. In a recent Canadian study the incidence rate of clinical mastitis was lower on organic farms than on conventional farms, but bulk milk SCC tended to be higher [20]. In a study carried out in the United Kingdom, no major differences were recorded between organic and conventional production [34]. In Sweden, where the requirements for organic production are similar as in Finland, cow composite milk SCC did not differ between the two production types [35]. Some studies reported similar results as here [36, 37]. In all studies cited, farm enrollment has been voluntary, which may create selection bias, possibly explaining discrepancies between the results. Factors explaining the higher cow milk SCC in organic farms could be avoidance of antibiotic treatments for mastitis, and lower milk yield in general compared with conventionally kept dairy cows, in addition to differences in management practices.

### Herd related factors associated with SCC, SCM and CSCM

Free-stalls with AMS were strongly associated with a higher milk SCC of individual cows and herds, as well as with a higher prevalence of SCM, while herd size had only a moderate effect in increasing milk SCC. In Finland, larger herds are mostly housed in free-stalls and often have AMS, and smaller herds are usually kept in tie-stalls. Good management and milking hygiene, as well as professionalism of the farmer, can decrease average milk SCC of the herd [5, 38, 39]. Lower milk SCC of cows in tie stalls may be due to individual care of the cows, including closer monitoring of udder health. Milking hygiene and mastitis detection have not been optimal with AMS milking, but improvements have been made to new models of AMS [40]. Recommended grouping of cows according to udder health status in free-stalls with AMS has not been feasible because the herds are relatively small in Finland [41]. The design and functionality of free-stalls built after 2010, as well as their management, differ substantially from those in free-stalls built in 2001. In a recent study from Finland, AMS was not a significant risk factor for pathogen-specific intramammary infection [33].

### Season and location of the farm

As previously reported, season had an impact on milk SCC [5, 42, 43]. SCC increased during late summer and was lowest during the cold and dry period in winter. Heat stress is known to affect milk SCC and milk production [44, 45], but may not be the most important factor under Finnish conditions. The summer season challenges ventilation systems in stalls and increasing warmth and humidity predispose cows to mastitis. According to Finnish legislation, cows in tie-stalls must graze at least 60 days during summer, but also free-stalls without grazing had a similar influence on SCC during late summer months (Fig. 2). Preventive measures for pathogen transmission in the summer season may be less efficient than during other seasons [46]. Using monthly test results would have provided more accurate knowledge on the seasonal effect, but those data were available for a very limited number of farms.

Location of the farm affected the results such that on farms located in West or South Finland both cow composite milk SCC and average SCC of the herd were higher than on farms in East and North Finland (Fig. 2). Accordingly, the risk for SCM and CSCM was lower in East and North Finland (Fig. 3). These findings may be linked to cultural, economic and management differences among farms in different parts of the country. Each area also has their own advisory services, which may slightly differ from each other.

### Quality of the study and possible bias

The Finnish dairy industry has undergone substantial structural changes during two decades. The total number of herds has decreased from 40,188 in 1991 to 11,256 in 2010, cows from 445,600 (1991) to 289,339 (2010) [2], and average herd sizes have increased. The proportion of herds included in the Finnish national health monitoring and milk recording system has also considerably increased during the three decades. The change from 1991 to 2001 is notable and may represent selection bias in the present study. The proportion of cows included in 1991 was much lower than in the other study years, which may also indicate selection bias. However, the number of cows remains sufficiently high that we considered it to be a representative sample of Finnish dairy cows belonging the national health monitoring and milk recording system. The number of cows in free-stalls associated with the Finnish national health monitoring and milk recording system was zero in 1991 and 2001, but we decided to include AMS stalls, and present the data as a supplementary file with separate models for 2001 and 2010 because we considered the information to be important. The databases used were large and could be used as a representative sample of Finnish dairy cows in each study year, which gives confidence for the study. This was enabled by the Finnish national health monitoring and milk recording system database. Including only cows for which there were four milk samples per year may have caused some selection bias, but on the other hand requiring more samples per year had caused a considerable loss of cows. Due to the excessive number of recordings, most of the tested variables were statistically significant and fitted in the final models, despite elimination of the cows with missing information. Associations between the tested variables and SCM and CSCM seemed moderate. Making blind selection from the dataset could have emphasized the effect of some of the tested variables, but considerable information would have been lost. As SCM is affected by a variety of factors, which we did not study here, we consider the moderate associations to be sufficiently accurate.

## Conclusions

This mastitis survey in Finland was the first that included cow composite milk SCC. Prevalence of CSCM was assessed for the first time. Prevalence of SCM in Finland decreased over the past three decades, from 22.3% (1991) and 20.1% (2001) to 19% (2010), the greatest reduction taking place during the first decade. Factors that possibly impacted the decreased risk for SCM are better breeding, management and housing of the dairy cows. Moreover, during the first decade national campaigns to improve udder health have contributed. The most significant cow and herd factors associated with SCM were increasing parity, Holstein breed, and free stalls with AMS. Prevalence of CSCM also decreased from 20.4% in 1991 to 16.1% in 2010; it was not much lower than that of SCM which indicates that most cows with SCM in fact had chronic mastitis. The Holstein breed and increasing parity were associated with CSCM. Prevalence of SCM has decreased but all the factors associated here with an increased milk SCC will continue to exist in the future. The Holstein breed will become more popular, and AMS is replacing conventional milking. These ongoing changes represent a challenge for dairy farm management and control of animal diseases such as SCM in the future. Regular surveys of mastitis prevalence would be useful to follow up on the situation and enable interventions to be made when needed.

## Additional files

**Additional file 1.** Effect of different factors on prevalence of subclinical mastitis (SCM) in Finland in years 2001 and 2010.

**Additional file 2.** Estimated effects of different factors on cow composite milk SCC (somatic cell count) in Finland in 2001 and 2010.

**Additional file 3.** Estimated effects of different factors on herd milk SCC (somatic cell count) in Finland in 2001 and 2010.
