# Supplementary material for: Prevalence of subclinical mastitis in Finnish dairy cows: changes during recent decades and impact of cow and herd factors
Source: Acta Vet Scand. 2017 Apr 20;59:22. doi: 10.1186/s13028-017-0288-x (PMC5397772; doi:10.1186/s13028-017-0288-x)
Supplement: Supplementary file 1 — Additional file 1. Effect of different factors on prevalence of subclinical mastitis (SCM) in Finland in years 2001 and 2010. [file 13028_2017_288_MOESM1_ESM.pdf]

Additional file 1 Effect of different factors on prevalence of subclinical mastitis (SCM) in Finland in years 2001 and 2010

			99.9 % CL		2001	2001 99.9 % CL		2010	2010 99.9 % CL	
	Comparison	Odds ratio	Lower	Upper	Odds ratio	Lower	Upper	Odds ratio	Lower	Upper
Year	2001 vs. 2010	1.126	1.099	1.153						
Parity	1 vs. 2	0.746	0.732	0.760	0.763	0.740	0.786	0.729	0.713	0.745
	1 vs. 3	0.526	0.516	0.537	0.566	0.548	0.584	0.490	0.478	0.501
	1 vs. ≥4	0.392	0.385	0.400	0.442	0.428	0.455	0.348	0.341	0.356
	2 vs. 3	0.706	0.692	0.719	0.742	0.719	0.765	0.671	0.657	0.686
	2 vs ≥4	0.526	0.517	0.535	0.579	0.562	0.596	0.478	0.468	0.488
	3 vs. ≥4	0.745	0.731	0.760	0.781	0.757	0.805	0.712	0.696	0.728
Breed	Ayrshire vs. Holstein	0.771	0.754	0.789						
	Ayrshire vs. Other breeds	0.892	0.810	0.982						
	Holstein vs. Other breeds	1.157	1.050	1.275						
Herd size	Average herd size	1.002	1.001	1.002						
Season	Jan - March vs. Apr - June	0.900	0.879	0.922						
	Jan - March vs. July - Sept	0.694	0.678	0.711						
	Jan - March vs. Oct - Dec	0.815	0.796	0.835						
	Apr - June vs. July - Sept	0.771	0.754	0.790						
	Apr - June vs. Oct - Dec	0.906	0.885	0.928						
	July - Sept vs. Oct - Dec	1.175	1.147	1.202						
Region	Southern vs Western	1.025	0.994	1.057						
	Southern vs Eastern	0.899	0.865	0.933						
	Southern vs Northern	0.865	0.839	0.891						
	Western vs Eastern	0.877	0.847	0.908						
	Western vs Northern	0.844	0.822	0.866						
	Eastern vs Northern	0.962	0.930	0.995						

Mixed effects logistic regression model of SCM and related factors (observations used 1,033,602). SCM was defined as cow composite milk SCC (somatic cell count) ≥200,000 cells/ml in one test milking of the year. Interactions between years and variables are presented separately in columns. OR (odds ratio), 99.9% CL (confidence limit).
