# Supplementary material for: Prevalence of subclinical mastitis in Finnish dairy cows: changes during recent decades and impact of cow and herd factors
Source: Acta Vet Scand. 2017 Apr 20;59:22. doi: 10.1186/s13028-017-0288-x (PMC5397772; doi:10.1186/s13028-017-0288-x)
Supplement: Supplementary file 2 — Additional file 2. Estimated effects of different factors on cow composite milk SCC (somatic cell count) in Finland in 2001 and 2010. [file 13028_2017_288_MOESM2_ESM.pdf]

Additional file 2 Estimated effects of different factors on cow composite milk SCC (somatic cell count) in Finland in 2001 and 2010

					2001			2010		
			99.9% CI			99.9% CI			99.9% CI	
Factor		Estimate	Lower	Upper	Estimate	Lower	Upper	Estimate	Lower	Upper
Year					88.4	86.9	89.9	79.2	78.1	80.4
Number of parity	1	61.2	60.4	62.1	66.1	64.9	67.4	56.7	55.9	57.6
	2	74.0	73.0	75.0	79.8	78.3	81.2	68.6	67.6	69.7
	3	95.3	94.0	96.6	100.2	98.3	102.2	90.6	89.2	92.0
	≥4	113.5	112.0	115.0	115.3	113.2	117.6	111.7	109.9	113.4
Breed	Ayrshire	76.8	76.0	77.6						
	Holstein	93.4	92.4	94.4						
	Other breeds	81.7	79.7	83.7						
Stall-type	Tie-stall	77.2	76.2	78.1						
	Free-stall	80.3	79.3	81.4						
	Free-stall (AMS)	94.5	93.0	95.9						
Type of production	Organic	87.5	85.7	89.3	94.2	91.4	97.2	81.2	79.2	83.4
	Conventional production	80.0	79.3	80.7	82.9	82.0	83.7	77.2	76.6	77.9
Region	East	81.2	80.1	82.2	87.0	85.4	88.6	75.7	74.5	76.9
	North	76.8	75.8	77.8	79.7	78.2	81.1	74.1	73.0	75.2
	South	87.0	85.8	88.3	92.0	90.2	93.9	82.3	80.9	83.7
	West	90.3	89.2	91.5	95.6	93.9	97.4	85.3	84.1	86.6

The estimated effects (multivariate ANOVA model) of the different pre-determined factors on composite milk SCC of cows included in the Finnish National health monitoring and milk recording system in 2001 and 2010 (observations used 1,033,602). Interactions between years and variables are presented separately in columns. All the included factors were tested statistically significant with Type III tests for fixed effects.
