# Supplementary material for: Prevalence of subclinical mastitis in Finnish dairy cows: changes during recent decades and impact of cow and herd factors
Source: Acta Vet Scand. 2017 Apr 20;59:22. doi: 10.1186/s13028-017-0288-x (PMC5397772; doi:10.1186/s13028-017-0288-x)
Supplement: Supplementary file 3 — Additional file 3. Estimated effects of different factors on herd milk SCC (somatic cell count) in Finland in 2001 and 2010. [file 13028_2017_288_MOESM3_ESM.pdf]

Additional file 3 Estimated effects of different factors on herd milk SCC (somatic cell count) in Finland in 2001 and 2010

		99.9% CI		
Comparison	Estimate	Lower	Upper	P-value
2001 vs. 2010	1.274	1.085	1.497	<.001
Average parity of the herd	0.965	0.941	0.989	<.001
Herd milk yield (100 kg)	0.991	0.990	0.992	<.001
Herd milk yield (100 kg, 2001)	0.989	0.987	0.990	<.001
Herd milk yield (100 kg, 2010)	0.993	0.991	0.994	<.001
Average herd size	1.005	1.004	1.006	<.001
Average herd size (2001)	1.007	1.005	1.009	<.001
Average herd size (2010)	1.003	1.002	1.004	<.001
Free-stall (AMS) vs. Free-stall	1.138	1.052	1.231	<.001
Free-stall (AMS) vs. Tie-stall	1.181	1.090	1.280	<.001
Free-stall vs. Tie-stall	1.038	0.998	1.079	0.014
South vs. West	1.002	0.963	1.042	0.909
South vs. North	1.092	1.046	1.139	<.001
South vs. East	1.106	1.063	1.151	<.001
West vs. North	1.090	1.055	1.126	<.001
West vs. East	1.104	1.072	1.137	<.001
North vs. East	1.013	0.981	1.046	0.301

The estimated effects (multivariate ANOVA model) of the different pre-determined factors on the average milk SCC of herds included in the Finnish National health monitoring and milk recording system in 2001 and 2010 (observations used 11,991). Interactions between years and variables are presented on separate lines. All the included factors were tested statistically significant with Type III tests for fixed effects. For detailed description of variables please see Table 1.
